# Editorial: Benefit and risk on drug-drug interactions in infections

**DOI:** 10.3389/fmicb.2025.1600984

**Published:** 2025-04-24

**Authors:** Hideo Kato, Nobuhiro Asai, Mao Hagihara

**Affiliations:** ^1^Department of Pharmacy, Mie University Hospital, Tsu, Japan; ^2^Division of Clinical Medical Science, Department of Clinical Pharmaceutics, Mie University Graduate School of Medicine, Tsu, Japan; ^3^Department of Clinical Infectious Diseases, Aichi Medical University, Nagakute, Japan; ^4^Department of Pathology, University of Michigan, Ann Arbor, MI, United States; ^5^Department of Molecular Epidemiology and Biomedical Sciences, Aichi Medical University Hospital, Nagakute, Japan

**Keywords:** drug-drug interaction, antibiotics, antifungals, antivirals, concomitant medications

The term “drug-drug interactions” (DDIs) refers to changes in a medication's toxicity or effectiveness due to the concurrent administration of another medication. This may occur often. Since clinically relevant DDIs are mainly associated with the absorption, distribution, metabolism, and excretion processes of each medication in the body, along with the interactions between different medications in the body, an in-depth understanding of the pharmacokinetic or pharmacodynamic processes can enhance antimicrobial effects, prevent the emergence of antimicrobial resistance, and reduce adverse events. However, information on DDIs is insufficient in clinical trials, and the process of validating potential DDIs one by one through biological experiments is time-consuming. On the other hand, in clinical settings, there are rare opportunities to encounter cases that were impacted by DDIs. This Research Topic includes four manuscripts reporting on the effects and adverse effects of DDIs based on data from clinical settings.

Non-tuberculous mycobacterial pulmonary disease (NTM-PD) is a refractory chronic respiratory infectious disease that occurs in patients with structural pulmonary abnormalities. Multi-drug therapy is the standard of care for NTM-PD. However, this treatment is frequently associated with adverse events due to DDIs, leading to the discontinuation of the standard treatment. Takeda et al. provided an overview of DDIs that can occur with NTM-PD multidrug therapy. Macrolides are crucial in the treatment of NTM-PD. Although ethambutol (EB) is an essential drug to suppress macrolide resistance, adjustments such as intermittent dosing and low-dose EB administration should be considered to avoid interruptions in EB treatment due to adverse events. The effect of rifampicin is limited, and its discontinuation is considered when concomitant use is difficult because of DDIs. In contrast, clofazimine regimens remain controversial because of the limited results in clinical trials. Amikacin liposome inhalation suspension (ALIS) has no impact on DDIs, whereas amikacin resistance occurs in 10% of cases after ALIS administration. This study contributes to efforts to optimize DDIs for the treatment of NTM-PD.

Letermovir (LET) is a novel antiviral agent for cytomegalovirus (CMV) prophylaxis in renal transplant patients. LET undergoes oxidative metabolism by cytochrome P450 (CYP)-3A4. LET exhibits both inhibition and weak induction of CYP3A4. Tacrolimus (TAC) is administered as an immunosuppressant in renal transplant patients. TAC is metabolized by CYP3A4. Therefore, the effects of LET on CYP3A4 and the metabolism of TAC by both CYP3A4 and CYP3A5 would be influenced by potential DDIs in their combination. However, their combined effects in Japanese renal transplant patients remain unclear. Maruyama et al. investigated the effects of LET on TAC pharmacokinetics in Japanese renal transplant patients via physiologically based pharmacokinetic (PBPK) modeling. Co-administration of LET with TAC significantly increased TAC exposure in the Japanese post-renal transplant population; the effect varied depending on the CYP3A5 genotype. Therefore, when LET is co-administered with TAC for Japanese renal transplant patients, TAC dosage should be reduced by ~57%−65% depending on the CYP3A5 genotype to maintain its therapeutic efficacy. This study provides insights into the development of effective immunosuppressive strategies for Japanese renal transplant patients.

Isavuconazole is a new extended-spectrum triazole for the treatment of invasive aspergillosis. Isavuconazole is a moderate inhibitor of CYP3A4 with fewer DDIs than voriconazole. Therefore, metabolic profiles of various CYP3A4 substrates have been reported with isavuconazole combination therapy. However, despite the need for a transition between azole antifungals due to toxicity in the clinical setting, reports on changes in the blood cyclosporine levels when transitioning from voriconazole to isavuconazole are limited. Shiraishi et al. illustrated one case showing the effect of switching from voriconazole to isavuconazole on blood cyclosporine levels. In addition, a PBPK model simulation was conducted to assess the potential interactions between isavuconazole and cyclosporine and between voriconazole and cyclosporine. Finally, the adverse events associated with cyclosporine in combination with voriconazole or isavuconazole were investigated using the FDA Adverse Event Spontaneous Reporting System (FAERS) database. In one case, cyclosporine blood levels decreased by more than half after switching from voriconazole to isavuconazole. Considering the inhibitory effects on the gastrointestinal tract, a PBPK analysis estimated that isavuconazole increased the area under the curve (AUC) and Cmax of cyclosporine by 1.48-fold and 1.84-fold, respectively, although assuming no change in gastrointestinal metabolism, these effects were minimal. The FAERS database included 9,144 reports of cyclosporine and 174 reports of cyclosporine with voriconazole but none with isavuconazole. The interaction of isavuconazole with cyclosporine was weaker than that with voriconazole. Maintaining a 2 h dosing interval between isavuconazole and cyclosporine may minimize gastrointestinal drug interactions.

The hybrid FowlTα1 peptide represents a biomolecule synthesized from Fowlicidins (Fowl) and Thymosin α1 (α1). This peptide exhibits remarkable anti-inflammatory and antimicrobial properties. Ahmad et al. investigated the supplemental effects of this peptide in interacting with lipopolysaccharides (LPS). In LPS-stimulated macrophages, the hybrid FowlTα1 peptide significantly reduced the release of nitric oxide, tumor necrosis factor-alpha, interleukin-6 (IL-6), and IL-1β in a dose-dependent manner and displayed robust antimicrobial activity against Escherichia coli compared to conventional antibiotics. The findings of the study highlight the potential of FowlTα1 peptide as a novel therapeutic agent for antimicrobial, anti-inflammatory, and anti-endotoxin applications.

In conclusion, this Research Topic provides valuable knowledge and information on the benefits and risks of DDIs in infections. Understanding DDIs can help develop effective strategies to enhance drug efficacy and to prevent the emergence of bacterial drug resistance.

